# Rater agreement of visual lameness assessment in horses during lungeing

**DOI:** 10.1111/evj.12385

**Published:** 2015-02-02

**Authors:** M. Hammarberg, A. Egenvall, T. Pfau, M. Rhodin

**Affiliations:** ^1^ Department of Clinical SciencesSwedish University of Agricultural SciencesUppsalaSweden; ^2^ Department of Veterinary Clinical SciencesThe Royal Veterinary CollegeUniversity of LondonHatfieldUK

**Keywords:** horse, agreement, circle, inter‐rater, lameness evaluation, lungeing

## Abstract

**Reasons for performing study:**

Lungeing is an important part of lameness examinations as the circular path may accentuate low‐grade lameness. Movement asymmetries related to the circular path, to compensatory movements and to pain make the lameness evaluation complex. Scientific studies have shown high inter‐rater variation when assessing lameness during straight line movement.

**Objectives:**

The aim was to estimate inter‐ and intra‐rater agreement of equine veterinarians evaluating lameness from videos of sound and lame horses during lungeing and to investigate the influence of veterinarians’ experience and the objective degree of movement asymmetry on rater agreement.

**Study design:**

Cross‐sectional observational study.

**Methods:**

Video recordings and quantitative gait analysis with inertial sensors were performed in 23 riding horses of various breeds. The horses were examined at trot on a straight line and during lungeing on soft or hard surfaces in both directions. One video sequence was recorded per condition and the horses were classified as forelimb lame, hindlimb lame or sound from objective straight line symmetry measurements. Equine veterinarians (n = 86), including 43 with >5 years of orthopaedic experience, participated in a web‐based survey and were asked to identify the lamest limb on 60 videos, including 10 repeats. The agreements between (inter‐rater) and within (intra‐rater) veterinarians were analysed with κ statistics (Fleiss, Cohen).

**Results:**

Inter‐rater agreement κ was 0.31 (0.38/0.25 for experienced/less experienced) and higher for forelimb (0.33) than for hindlimb lameness (0.11) or soundness (0.08) evaluation. Median intra‐rater agreement κ was 0.57.

**Conclusions:**

Inter‐rater agreement was poor for less experienced raters, and for all raters when evaluating hindlimb lameness. Since identification of the lame limb/limbs is a prerequisite for successful diagnosis, treatment and recovery, the high inter‐rater variation when evaluating lameness on the lunge is likely to influence the accuracy and repeatability of lameness examinations and, indirectly, the efficacy of treatment.

## Introduction

Lameness is a frequent reason for decreased performance in horses and hence for veterinary intervention 1, 2. When evaluating lameness it is common practice to study the horse both on the straight and during lungeing, since it has been suggested that circling a horse in trot may enhance low grade lameness 3, 4. However, a number of factors may complicate the evaluation of lameness in horses during lungeing. Studies have shown that vertical movement symmetry is systematically affected when trotting in a circle and asymmetry is most pronounced on the inner hindlimb 5, 6. Also, circle size and speed will affect movement symmetry 7, 8. Further, compensatory lameness mechanisms are present during lungeing, especially for primary hindlimb lameness 6.

Even though objective systems quantifying movement asymmetries observable during lameness evaluations are emerging, it is still standard practice to perform the evaluation by visual inspection only. Scientific studies have shown that low agreement exists among veterinarians when visually evaluating whether a limb is lame or not in horses trotting in a straight line, on treadmills or over ground 8, 9, 10, 11. The agreement between experienced equine clinicians evaluating clinically lame horses on the straight at the trot in real time was studied by Keegan *et al*. 10. The clinicians agreed on whether or not a limb was lame in 93% of the cases if the horse had a lameness of >1.5 degree (AAEP scale) but only in 62% of the cases when the lameness was ≤1.5 degree. The agreement did not increase after the veterinarians had performed a full lameness evaluation in which flexion tests and lungeing were allowed. Since lungeing is a common part of the lameness work‐up and some lamenesses are only visible during lungeing, it is of interest to investigate the agreement of a large number of equine veterinarians when visually evaluating lameness in horses during lungeing.

The purpose of this study was to quantify inter‐ and intra‐rater agreement among equine veterinarians visually scoring lameness in horses during lungeing. In addition, the influences of the veterinarians’ experience and the degree of objectively measured movement asymmetries of the horses on the rater agreement were evaluated. The hypothesis was that the result of visual lameness evaluation varies greatly between and within veterinarians and that rater agreement improves with increased experience and increased degree of movement asymmetry.

## Material and methods

### Horses

A total of 23 riding horses (17 Warmbloods and 6 ponies; 11 geldings, 11 mares and one stallion from 9 stables) were included in this cross‐sectional study. The mean withers height was 161 cm (range 135–170 cm) and the mean age 11 years (range 4–21 years). The horses were used for dressage (n = 9), showjumping (n = 1) or both (n = 13) at basic (n = 20) or intermediate (n = 3) level. Two of these horses were included when they participated in a study where lameness was induced 6 by tightening a screw to cause pressure pain to the sole 12. The motion patterns of all horses were measured with an inertial sensor based system (see below) at trot in‐hand on the straight and during lungeing in both directions (circle approximately 8–10 m in diameter) on hard gravel based or soft surfaces at the location where horses were stabled or at a competition. The horses with induced lameness were lunged with lameness induced on different limbs (one limb at a time). Horses were filmed from the outside of the circle using either a camera with digital video or high definition format on a tripod. The camera was not panned or tilted and pointed to the centre of the circle with the horse in view for the whole circle.

### Objective symmetry measurements

One accelerometer was taped to a head bumper attached to the poll and one accelerometer to the midline pelvis between the *tubera sacrales*. The sensitive axis of the single‐axis accelerometer was aligned with gravity (positive upwards). To determine timing of right/left fore‐ and hindlimb stance a gyroscopic transducer was strapped to the dorsal surface of the right forelimb pastern. Real‐time sensor data were digitally sampled (8‐bit) at 200 Hz. Data were collected and analysed in software, custom‐written in Delphi^a^ and Matlab^b^. Detailed descriptions can be found in Keegan *et al*. 13, 14.

Double‐integrated vertical head and pelvic accelerations were processed using an integration error correction algorithm 15. From each stride, 2 local maxima and minima were located in the signal. Differences in maximum head (HDmax) and pelvis (PDmax) displacement after right and left stance phases and differences in minimum head (HDmin) and pelvis (PDmin) displacement during right and left stance phases were calculated per stride 13, 15, 16.

### Objective lameness definition

The horses were defined as sound, forelimb lame or hindlimb lame from HDmin and PDmin values from straight line measurements. The criterion for straight line lameness in the current study was a mean difference in head or pelvic excursion consistently outside the normal ranges: ±6 mm for HDmin and/or HDmax and ±3 mm for PDmin and/or PDmax with standard deviations less than their respective means 13. For a left sided lameness, HDmin or PDmin were negative and for a right sided lameness, positive. One horse (without induced lameness) was found lame on 2 limbs and was categorised as primary hindlimb lame based on the fact that the lameness was ipsilateral 6 (even though it could be multilimb lameness and not a compensatory lameness mechanism).

### Videos

A total of 50 videos, one lungeing direction/video, were selected based on video quality and 23 videos showed horses during lungeing to the right and 27 to the left, of which 20/30 were lunged on a hard/soft gravel based surface.

To provide videos on horses with different types of lameness, the straight line objective lameness evaluations were used for the selection. Horses defined as forelimb lame (from the straight line objective lameness evaluation) were shown in 21 videos, as hindlimb lame in 19 and as sound in 10 videos. Of these videos, 10 (4 hindlimb lame, 4 forelimb lame and 2 sound) were repeated for intra‐rater agreement evaluation. The number of repeated videos was limited to make evaluator/observer compliance possible. The 23 horses were shown between one and 4 times each during different conditions (lungeing directions, different ground surfaces, different limbs of lameness induction) in the questionnaire. The 2 horses with induced lameness were shown in 2 and 6 videos, respectively, and one of these was repeated.

### Objective degree of movement symmetry during lungeing

Based on the results from the objective symmetry measurements of the horses during lungeing in the selected videos, the horses were categorised into one of 4 (0–3) asymmetry categories for fore‐ and hindlimbs, respectively. For the forelimbs (FL) the absolute value (mm) for HDmin was used (FL0 <6 mm; FL1 6–12 mm; FL2 12–18 mm; FL3 >18 mm) and for the hindlimb the absolute value for PDmin (HL; HL0 <3 mm; HL1 3–6 mm; HL2 6–9 mm; HL3 >9 mm) categorisation, taking simple multiples of the straight cutoff values. Two videos were excluded from κ analysis of the influence of the different symmetry categories during lungeing owing to missing data from the motion analysis.

### Participants

In an effort to include as many practising equine veterinarians in Sweden as possible, an invitation to participate anonymously in the survey was sent by e‐mail to the members of the Swedish Equine Veterinary Association, veterinarians working on Swedish racetracks, and other equine veterinarians who were known to the authors. Reminders were e‐mailed 10 days after the invitation and one week before the closing date of the survey. The participants were given instructions by e‐mail on how to participate in the survey (Supplementary Item 1). Experience category was defined as more experienced (>5 years of experience of equine orthopaedic work and at the time of the survey doing 100% equine work) and less experienced equine veterinarians.

### Web‐based questionnaire

Videos (n = 60, including the 10 repeats, 20 s) of sound and lame horses during lungeing were presented in LimeSurvey^c^ 1,9 +, a software for web‐based surveys. The survey was divided into 4 sections: 1) instructions; 2) information about the participant, 8 questions (Supplementary Item 1); 3) lameness evaluation; and 4) feedback to the participant. In the lameness evaluation section, the videos, in Flash Video format, of horses trotting in a circle were presented. The video sequences could be watched an unlimited number of times before scoring and viewed in full or 10 × 15 cm sized screens. The participant was first asked to comment whether the quality of the video was considered sufficient for evaluation of the movement of the horse. If the quality of the video was considered adequate the participant was asked to evaluate the movement of the horse. All 4 limbs were evaluated from the trotting sequence and scored 0–5, a scale permitting grading independently for each gait and situation, where 0 represented soundness and 5 represented a nonweightbearing lameness, and scores in half units were allowed. This was followed by the question: ‘Which limb would you start to examine in the case of a lameness examination?’ and the given options were ‘right front’ (RF), ‘left front’ (LF), ‘right hind’ (RH), ‘left hind’ (LH) or ‘the horse does not need further investigation’ (sound). The participant was then given 3 options: 1) finalise questionnaire; 2) save answers and continue the questionnaire on a later occasion; or 3) save answer and continue to the next question. A saved answer could not be changed and all participants evaluated the videos in the same order.

In the feedback section, which was included to motivate participation, the participants were given the option to view videos again and the result of the objective lameness evaluation for straight line was presented. Information on whether the lameness was induced, if the video sequence was a repeat and anonymous feedback of how the previous participants answered were also given (data not shown). When the questionnaire was finished, the participant was e‐mailed his/her own results and they were offered the option to look at the videos once more. The survey was open for a total of 33 days. No actions were taken to prevent participants from communicating with each other. The time for completing the survey was registered for each participant.

### Data analysis

Fleiss’ κ (κ) was used to demonstrate inter‐rater agreement overall and by experience category for all unique videos (the second evaluation of the repeated videos was excluded from the analysis). Results for the 5 lameness options are presented in percentages for each video. These analyses were also made on a subset of different video categories (where the majority of the veterinarians selected: no further investigation [sound]; lameness [either fore‐ or hindlimb]; forelimb lameness; or hindlimb lameness), and according to the asymmetry categories (FL0, HL0, FL3, and HL3).

Cohen's κ was used to illustrate the distribution of intra‐rater agreement for the 10 repeated videos (i.e. the ‘video pairs’). The agreement for both κ statistics was considered poor for κ≤0.3, acceptable for κ = 0.31–0.5, good κ = 0.51–0.8 and excellent for κ>0.8.

## Results

### Participants

The survey was sent to 462 veterinarians, 194 of whom started and 94 completed the survey. Eight veterinarians were excluded for suspected noncompliance with the survey by consistently suggesting that the limb to be examined first was not the limb that they had identified as lame. Of the remaining 86 veterinarians (18.6% effective response rate), 43 were defined as experienced and 43 less experienced equine orthopaedic clinicians, the gender distribution was 59/27 women/men (experience category by gender; experienced 27/16; less experienced 32/11), 50 worked in specialist centres, 27 in ambulatory practice and 5 combined both. There was one veterinarian in mixed practice, one official racetrack veterinarian, one combined reproduction plus work as official racetrack veterinarian and one did not state the type of practice. The distribution of years of experience and part of the workday dedicated to equine orthopaedics are shown in Table 1. Time of completion of the questionnaire was <24 h for 18 participants but the median time was 5.5 (range 1–33) days.

**Table 1:** Participants’ equine orthopaedic experience

Part of orthopaedic work (%)	Years of equine orthopaedic experience				Total
	0–5	5–10	10–15	>15	
100	0	0	0	1	1
80	4	6	5	10	25
60	6	3	2	8	19
40	1	2	1	3	7
20	2	1	0	6	9
<20	17	2	1	5	25
Total	30	14	9	33	86

### Videos

The most common comments as to why the videos could not be evaluated were: the horse was trotting too fast/slow; the size of the circle was too big/small; and the general quality of the video was not adequate. Of all the 60 videos shown including the repeats, the evaluations of 3 videos were removed from further analysis because the video quality was considered inadequate by 40, 26 and 14% of the participants, respectively. The participants considered the remaining 57 videos of adequate quality in 96.5% of the evaluations. Each video was evaluated by (median) 84 veterinarians (Supplementary Item 2).

### Asymmetries in the motion pattern during lungeing

**Figure 1:**
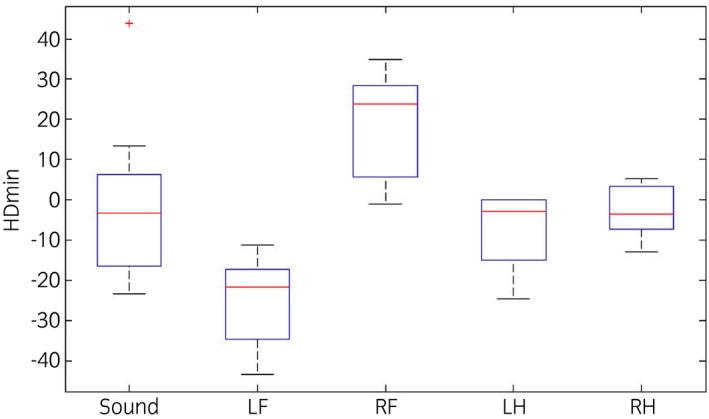
Box plots of the difference in minimum head displacement (mm) during right and left stance phases (HDmin) from the objective motion analysis during lungeing for the horses on the videos where the majority of the participants selected: no further investigation (Sound), left fore (LF), right fore (RF), left hind (LH) or right hind (RH) lameness. A negative value indicates a left and a positive value a right forelimb asymmetry.

**Figure 2:**
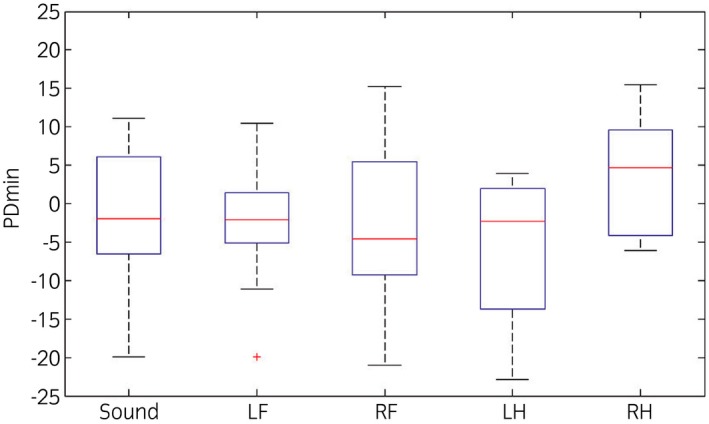
Box plots of the difference in minimum pelvic displacement (mm) during right and left stance phases (PDmin) from the objective motion analysis during lungeing for the horses on the videos where the majority of the participants selected: no further investigation (Sound), left fore (LF), right fore (RF), left hind (LH) or right hind (RH) lameness. A negative value indicates a left and a positive value a right hindlimb asymmetry.

Numbers of videos of horses with the different fore‐ and hindlimb asymmetry categories during lungeing (n = 45) are shown in Supplementary Item 3. The HDmin and PDmin values for the horses on the videos where the majority of the participants selected; no further investigation (sound), left fore (LF), right fore (RF), left hind (LH) or right hind (RH) lameness are shown in Figures 1 and 2.

### Visual lameness scoring

The participants graded the lameness for the most lame limb ≤1.5 degrees in 68% and >1.5 degrees in 32% of the evaluations.

### Inter‐rater agreement

K values and 95% confidence intervals (95% CI) for the inter‐rater agreement, for all participants, on the answer on the question ‘which limb would you start to examine in the case of a lameness examination’ with the given 5 options RF, LF, RH, LH or sound, are presented in Table 2. The mean, standard deviation, minimal and maximal percentages were calculated for the option with the highest agreement. The calculations were also made for 8 subset categories (Table 2). The inter‐rater agreement for the less experienced equine orthopaedic clinicians was poor (κ = 0.25, 95% CI 0.247–0.256) and acceptable for the experienced equine orthopaedic clinicians (κ = 0.38, 95% CI 0.376–0.385) when evaluating all 47 videos (repeats excluded). In 9 (15.8%) of the 57 videos the majority of the experienced group disagreed with the majority of the less experienced group on which limb to start to examine (the most lame limb).

**Table 2:** Inter‐rater agreement (κ) and percentages for the option with the highest agreement for all and subsets of the videos

Category	Videos	κ	95% CI		Highest agreement (%)			
	(n)		lower	upper	mean	s.d.	min	max
All	47	0.31	0.307	0.312	61	17.5	30	93
Sound	15	0.08	0.077	0.086	57	17.5	37	93
Lame	32	0.31	0.309	0.315	62	17.6	30	91
FL lame	22	0.33	0.326	0.334	69	12.7	41	91
HL lame	10	0.11	0.104	0.114	47	12.1	30	69
**FL 0**	13	0.19	0.183	0.193	53	13.0	36	76
**HL 0**	11	0.30	0.297	0.307	59	8.1	36	88
**FL 3**	23	0.33	0.328	0.335	64	17.9	36	87
**HL 3**	16	0.31	0.306	0.313	58	19.2	30	88

Inter‐rater agreement (κ and 95% confidence interval) for all 86 veterinarians’ evaluation of n videos. The mean, standard deviation, minimal and maximal percentages for the option with the highest agreement are presented. The categories were based on what the majority selected: no further investigation (sound); lame (either fore‐ or hindlimb); forelimb lameness (FL lame); or hindlimb lameness (HL lame). The videos were also categorised according to the asymmetry categories (Supplementary Item 3): FL 0; HL 0; FL 3; and HL 3.

### Intra‐rater agreement

Mean Cohen's κ for the intra‐rater agreement was 0.52 with percentiles P10 = 0.21, P50 = 0.57 and P90 = 0.84). Of the 72 participants who evaluated 9 or 10 video pairs (the other 14 participants evaluated <9 video pairs) the highest intra‐rater agreement was κ = 0.87.

### Evaluations of videos from horses with induced lameness

Videos on induced forelimb lameness (n = 3) were evaluated correctly by 74% (mean) of the evaluators (range 72–88%) and for videos on hindlimb lameness (n = 5) by 37% (mean) of the raters (range 22–69%; Table 3).

**Table 3:** Evaluations of 8 videos from horses with induced lameness

Direction	Induced	RF (%)	LF (%)	RH (%)	LH (%)	Sound (%)	Horse
L	*LF*	6.2	**75.3**	8.6	6.2	3.7	1
R	*LF*	4.9	**87.7**	3.7	2.5	1.2	1
R	*RF*	**72.0**	6.1	12.2	7.3	2.4	2
R	*LH*	3.5	**65.9**	7.1	22.4	1.2	1
L	*LH*	2.7	**68.0**	4.0	22.7	2.7	1
L	*LH*	9.3	**41.9**	5.8	**41.9**	1.2	2
R	*RH*	2.5	24.1	**40.5**	8.9	24.1	1
L	*RH*	0.0	2.4	**68.7**	20.5	8.4	1

Videos of 2 horses with induced lameness on left fore (*LF*), right fore (*RF*), left hind (*LH*) and right hindlimbs (*RH*) lunged in either left (L) or right (R) direction. The most lame limb (RF, LF, RH or LH) or sound chosen for each video by the participants in percentage are shown where bold indicates which limb the participants agreed on most.

## Discussion

These results indicate that visual lameness assessment of horses trotting in a circle has poor agreement for less experienced equine veterinarians and moderately acceptable agreement for experienced orthopaedic equine practitioners. The inter‐rater agreement was higher for the videos where the majority selected a forelimb (κ = 0.33) compared to hindlimb lameness (κ = 0.11) evaluation and lowest for the sound evaluation (κ = 0.08). For the forelimbs there was markedly higher agreement for more pronounced lameness. In a previous study, where 2–5 clinicians (85% of the evaluations were made by ACVS board‐certified surgeons) evaluated 131 horses live, inter‐rater agreement for deciding whether the horse was lame or sound while trotting in a straight line was κ = 0.44 (0.51 for the forelimbs and 0.36 for the hindlimbs) 10. The agreement was higher than in the current study, especially for detection of hindlimb lameness. This could be due to firstly the high proportion of less experienced participants in the current study and secondly the difficulties in evaluating hindlimb lameness on the lunge. Additionally, different lameness scales were used in the previous and current studies, which is of particular relevance when showing differences in agreement as a function of lameness grade. In the current study, the horses were assessed for lameness from the trotting sequence during lungeing, and each limb scored 0–5 using a scale permitting grading independently for each gait and situation (0 representing soundness and 5 representing non‐weightbearing lameness).

The majority of the participants evaluated the horse as sound in only 15 videos and the agreement ranged from 37–93% for the different videos. It might be more difficult to define soundness in horses during lungeing since the circling induces motion asymmetries also in sound horses that can be difficult to distinguish from lameness 6, 7. These small but measurable asymmetries are affected by both circle size, speed 7, 8 and probably the handedness of the horse.

### Sensitivity for visual lameness assessment

There is no ‘gold standard’ for lameness evaluation during lungeing and therefore HDmin and PDmin values from straight line measurements were used in order to provide a spectrum of different degrees of fore/hindlimb lame and sound horses even though the lameness *per se* could change when the horse was lunged. These head and pelvic motion variables have been used in previous studies to define fore‐ and/or hindlimb lameness 16, 17, 18.

We aimed to investigate the agreement between veterinarians because the true soundness/lameness status of most of the horses was not known and hence it is impossible to calculate the sensitivity and specificity for the visual lameness assessment. However, 8 of the videos (Table 3) were recorded from an experiment where lameness was induced (reversible hoof pressure in each limb, one at a time) and evaluated objectively 6. For 2 of these videos, the majority of the raters scored a sound limb as lame and hence the agreement for those selecting the correct lame limb will probably be even lower. For forelimb lameness, 3 videos were evaluated correctly by an average of 74% of the raters and for 5 videos on hindlimb lameness by a mean of 37% of the raters, suggesting that the sensitivity for forelimb lameness detection is higher (0.74) than for hindlimb lameness detection (0.37); although the range was wide for the latter, the number of observations was low in both groups and they were dependent, representing only 2 horses. The reason for the low number of correct answers for the hindlimb lameness could be that the compensatory ipsilateral forelimb asymmetry actually appeared more obvious for >65% of the raters for 2 of the videos (Table 3). This highlights the importance of understanding how the horse compensates its motion pattern to avoid painful loading or movement of a limb during lungeing 6. Also, the inner hindlimb asymmetry seen in sound horses trotting in a circle may influence hindlimb lameness, making it less visible depending on the lameness affecting the inside or outside limb on the circle.

### Objective measurements during lungeing

It is not possible to find clear cut offs between the vertical symmetry measurement values (HDmin and PDmin) between the groups of videos where the majority of the veterinarians agreed on lameness (for different limbs) or soundness (Figs 1, 2). Even within the group of horses that were considered ‘sound’ by the majority, movement symmetry varied considerably by up to 44 mm for head movement and 18 mm for pelvic movement (Supplementary Item 2). We do not know whether these horses were evaluated incorrectly and actually were lame or whether individual horses use different biomechanical mechanisms to produce the necessary forces to move in circles, owing to conformation, handedness or asymmetric training, which could be reasons for biological variation.

It might be difficult to define general thresholds for soundness for objective symmetry measurements on the lunge, but objective methods will be very useful when comparing movement symmetry before and after diagnostic analgesia or necessary when evaluating different treatments or rehabilitation programs in research on horses lame on the lunge.

### Benefits and limitations of the study

One of the practical difficulties with conducting a study of visual lameness assessment is to get a large number of veterinarians evaluating the same horses, at the same place ‘live’ rather than from videos. In this study a web‐based survey was used to enable a large number of veterinarians to evaluate the same horses. As many as 194 of the recipients started the survey, but only 94 completed and the low completion rate might be explained by the amount of time and effort needed to evaluate all videos. Only 18/86 participants concluded the survey within 24 h, and the median time to complete the survey was 5.5 days.

Advantages with videos include that specific sequences can be selected to ensure that the veterinarians evaluate the same sequence with the same circle size, and trotting speed and that the veterinarian has the chance to look at the video as many times as wanted. Disadvantages of video recordings, regarding the possibility to identify the lame limbs correctly, include the inability to change circle size, trotting speed or angle of view. The video method has been used in previous studies 8, 19, 20 and in the latter study, an intra‐rater (2 raters, including one very experienced) repeatability study of the lameness evaluation of 506 horses achieved 98% correlation. In the current study the intra‐rater agreement was good (median) κ = 0.52 with the highest value of 0.87. The reason for this lower agreement compared to the study by Greve and Dyson 20 is difficult to interpret, since both the severity of lameness and the situation where the horses were evaluated as lame (straight line, lungeing or ridden) could be different, but the very experienced clinician may contribute to the high correlation. Based on the low percentage of videos considered of inadequate quality the general quality of videos was considered to be good. A recurring comment to why a video was considered of inadequate quality was the trotting speed being either too low or too high, but in a recent study [8], it was concluded that trotting speed did not affect the agreement when 6 veterinarians evaluated lameness during lungeing. The circle size used in the current study may influence the results as different veterinarians may prefer or are used to evaluating lameness at a specific circle size. For some of the videos considered of inadequate quality and not evaluated by all the participants, the circle size was the reason. It should also be emphasised that the videos showing horses with induced lameness originated from only 2 horses, and attempts were not made to correct for this in the analysis. However, this will limit the capacity for the findings to be extrapolated to the real‐world situation.

## Conclusions

The results of the current study indicate that visual lameness assessment of horses during lungeing had a poor agreement for less experienced equine veterinarians and poor for all raters on hindlimb lameness and soundness. Since identification of the lame limb/limbs is a prerequisite for successful diagnosis, treatment and recovery, the high inter‐rater variation when evaluating lameness on the lunge is likely to influence the accuracy and repeatability of lameness examinations and consequently the prognosis after treatment.

## Authors’ declaration of interests

None of the authors has any financial or personal relationship that could inappropriately influence or bias the contents of the paper.

## Ethical animal research

The study has been approved by the Ethical Committee for Animal Experiments, Uppsala, Sweden (permission number C251/9) and informed consent for data collection was obtained from the horse owners prior to the study.

## Source of funding

Swedish‐Norwegian Foundation for Equine Research funded the study.

## Authorship

The study was designed by M. Rhodin, M. Hammarberg and A. Egenvall. Data collection and study execution were done by M. Hammarberg and M. Rhodin. All authors contributed to the data analysis and interpretation, to the preparation of the manuscript and approved the final version of the manuscript.

## Supporting information


**Supplementary Item 1:** The instructions and questions to the veterinarians participating in the survey.

**Supplementary Item 2:** Objective and subjective evaluation of the horses in the 47 videos (repeats excluded).

**Supplementary Item 3:** Number of videos (total 45) on horses with the different combinations of fore and hindlimb asymmetry categories during lungeing.

## Manufacturers’ addresses

^a^Borland Software Corp, Austin, Texas, USA.

^b^The Mathswork Inc, Natick, Massachusetts, USA.

^c^LimeSurvey Project Team, Hamburg, Germany.
