# Supplementary material for: Rater agreement of visual lameness assessment in horses during lungeing
Source: Equine Vet J. 2015 Feb 2;48(1):78–82. doi: 10.1111/evj.12385 (PMC4964936; doi:10.1111/evj.12385)
Supplement: Supplementary file 1 — Supplementary Item 1: The instructions and questions to the veterinarians participating in the survey. [file EVJ-48-78-s001.pdf]

Equine Veterinary Journal

Supplementary Item 1: Instructions to the participants before they started the survey

Lameness evaluation during lungeing

Welcome to participate in a web-based survey to evaluate lameness during lungeing. To participate you need to login by using your username and password.

Horses

The horses on the videos have been evaluated by Lameness Locator, (a sensor based system for lameness detection), on the straight and have been classified as sound or lame and the lame limb/limbs has been identified. Among participating horses some have induced lameness while others are ‘naturally’ lame. The horses are of different age, breed and use at varying levels. Some horses have a known lameness problem and some have been considered sound by the owner.

The survey

Before you start the lameness evaluation we ask you to answer some questions about you and your experience (see questions below). The reason for this information is to see how the participants´ education and experience influence the ability to evaluate lameness.

This survey contains 60 videos. Each video is 20 s in duration and you can watch it an unlimited number of times. Your task is to look at each video and evaluate if the horse is lame or not and identify the lame limb/limbs. You need to fill in a lameness score for each limb, 0 to 5, where 0 represents soundness and 5 represents a non-weight bearing lameness and scores in half units are allowed. All limbs need to be evaluated before continuing to the next question. When you have evaluated all limbs, we ask you to decide which limb you would start to examine in the case of a lameness examination, i.e. the lame limb you suggest as the primary lameness. If you for some reason cannot evaluate the lameness, choose the alternative ”not possible to evaluate”. If you choose this alternative, there is a box where you can comment on why it is not possible to evaluate the lameness.

Questions to the veterinarians participating in the survey.

**Questions**	Alternatives					
Gender	Female	Male	Confidential			
How long, in years, have you worked as a veterinarian?	0-5	5-10	10-15	> 15		
Do you have a post graduate degree, for example ‘Diplomate ECVS’, Swedish specialist degree in equine veterinary medicine?		Free text				
How much of your daily work is devoted to equine veterinary medicine?	< 20%	20 %	40 %	60 %	80%	100%
In what kind of practice do you work?	Ambulatory		Clinic	Other		
How much of your daily work is devoted to equine orthopaedics?	< 20 %	20%	40%	60%	80%	100%
How many years of experience do you have in equine orthopaedics?	0-5 yrs	5-10 yrs	10-15 yrs	> 15 yrs		
In which country and for how long did you study veterinary medicine?	Sweden 5.5 yrs			Other (free text)		
