# Supplementary material for: Rater agreement of visual lameness assessment in horses during lungeing
Source: Equine Vet J. 2015 Feb 2;48(1):78–82. doi: 10.1111/evj.12385 (PMC4964936; doi:10.1111/evj.12385)
Supplement: Supplementary file 2 — Supplementary Item 2: Objective and subjective evaluation of the horses in the 47 videos (repeats excluded). [file EVJ-48-78-s002.pdf]

Equine Veterinary Journal

Supplementary Item 2: Objective and subjective evaluation of the horses in the 47 videos (repeats excluded).

L/R	HDmin (mm)	SD	PDmin (mm)	SD	Asymmetry cathegory		RF (%)	LF (%)	RH (%)	LH (%)	Sound (%)	No evaluators
					fore	hind						
R	-11.5	7.20	-2.0	4.60	LF1	0	2.4	0.0	0.0	4.9	**92.7**	82
R	-21.5	12.70	10.5	2.52	LF3	RH3	8.3	**90.5**	0.0	0.0	1.2	84
L	-17.8	7.63	-2.4	2.33	LF3	0	7.0	**88.4**	2.3	1.2	1.2	86
L	27.6	8.53	-10.0	1.76	RF3	LH3	**88.2**	8.2	1.2	0.0	2.4	85
R	-43.3	19.15	3.4	3.87	LF3	LH1	4.5	**87.7**	3.7	2.5	1.2	81
L	34.8	21.54	-12.6	2.65	RF3	LH3	**85.7**	3.6	6.0	4.8	0.0	84
L	13.5	5.91	-6.9	3.28	RF2	LH2	7.1	1.2	6.0	2.4	**83.3**	84
R	-11.2	6.37	-1.5	3.16	LF3	0	9.4	**81.2**	2.4	7.1	0.0	85
L	23.8	10.54	-6.6	2.30	RF3	LH2	**78.9**	10.6	4.7	4.7	1.2	85
R	-2.5	6.37	2.3	2.32	0	0	5.8	0.0	8.1	10.5	**75.6**	86
L	-14.8	6.25	-5.1	2.06	LF3	LH1	7.0	**75.6**	7.0	5.8	4.7	86
L	-35.3	13.57	-11.1	2.95	LF3	LH3	6.2	**75.3**	8.6	6.2	3.7	81
L	5.3	9.74	7.4	3.59	0	RH2	6.0	3.6	7.1	8.3	**75.0**	84
R	-21.9	7.57	1.4	3.83	LF3	0	3.6	**73.8**	10.7	11.9	0.0	84
R	28.6	16.09	15.2	3.65	RF3	RH3	**72.0**	6.1	12.2	7.3	2.4	82
L	5.2	8.35	4.1	3.56	0	RH1	0.0	2.4	**68.7**	20.5	8.4	83
L	-30.0	19.39	-19.9	8.77	LF3	LH3	2.7	**68.0**	4.0	22.7	2.7	75
L	34.1	11.58	-21.0	2.71	RF3	LH3	**67.4**	5.8	7.0	19.8	0.0	86
R	-	-	-	-	-	-	**67.1**	10.6	2.4	7.1	12.9	85
R	-34.7	12.24	-4.9	2.89	LF3	LH1	3.5	**65.9**	7.1	22.4	1.2	85
R	8.6	8.81	-4.6	2.41	RF2	RH3	**65.1**	4.8	10.8	8.4	10.8	83
L	-5.0	7.62	-6.1	2.40	0	LH2	1.2	5.9	**62.4**	18.8	11.8	85
R	-23.3	16.89	8.9	2.69	LF3	RH2	3.5	27.1	3.5	3.5	**62.4**	85
R	-7.7	18.49	9.8	4.50	RF1	RH3	1.2	8.6	14.8	13.6	**61.7**	81
L	4.8	5.76	-6.9	2.74	0	LH2	**60.2**	1.2	7.2	10.8	20.5	83
L	-3.4	36.96	-5.5	3.01	0	LH1	12.7	7.6	12.7	10.1	**57.0**	79
R	-0.1	5.42	3.9	2.34	0	RH1	4.9	0.0	22.0	**53.7**	19.5	82
L	-8.1	12.25	-19.9	3.01	LF1	LH3	8.2	3.5	7.1	28.2	**53.0**	85
R	4.8	5.28	5.8	1.88	0	RH1	**52.4**	4.8	8.3	2.4	32.1	84
L	12.9	7.56	-0.7	1.83	RF2	0	10.7	10.7	21.3	6.7	**50.7**	75
L	43.8	25.17	-18.0	3.78	RF2	RH3	6.5	5.2	16.9	20.8	**50.7**	77
R	25.2	6.79	7.2	3.07	RF3	RH2	**48.8**	10.5	16.3	20.9	3.5	86
L	-17.3	9.05	-1.7	2.36	LF3	0	5.1	**48.7**	16.7	14.1	15.4	78

R	3.3	3.19	5.3	2.69	0	RH1	9.5	3.6	**45.2**	13.1	28.6	84
R	-0.9	17.22	-2.9	2.78	0	0	**45.2**	3.6	3.6	19.1	28.6	84
R	-5.5	4.28	-4.5	3.34	0	LH1	3.8	2.5	15.0	**45.0**	33.8	80
L	-13.0	7.75	-4.1	2.90	LF3	LH1	1.2	20.0	**44.7**	10.6	23.5	85
L	-18.9	10.30	11.1	3.17	LF3	RH3	2.4	29.8	14.3	9.5	**44.1**	84
L	-18.6	9.25	1.7	2.76	LF3	0	1.2	2.4	**41.0**	12.1	**43.4**	83
L	-24.5	18.22	-22.9	2.27	LF3	LH3	9.3	**41.9**	5.8	**41.9**	1.2	86
L	18.7	6.95	4.2	2.17	RF3	RH1	**41.0**	7.2	22.9	22.9	6.0	83
L	-2.2	8.46	15.5	2.69	0	RH3	2.5	24.1	**40.1**	8.9	24.1	79
R	6.5	8.83	-13.7	2.68	RF1	LH3	3.5	1.2	34.9	20.9	**38.4**	85
R	-18.1	13.09	-2.8	3.35	LF3	0	13.4	15.9	25.6	7.3	**37.8**	82
L	3.6	6.14	-2.2	1.74	0	0	29.4	8.2	7.1	18.8	**36.5**	85
R		-	-	-	-	-	25.0	7.5	8.8	**33.8**	25.0	80
L	-7.3	7.20	9.6	2.30	LF1	RH3	4.8	20.2	**29.8**	26.2	18.6	84

Measurements of mean HDmin and PDmin and standard deviation (s.d.) of the horses during lungeing in left (L) or right (R) direction. The categorisation of the fore- and hindlimb asymmetries 0-3 for left fore (LF), right fore (RF), left hind (LH) and right hind (RH) respectively. The lamest limb chosen for each video by the participants in percentage are shown in the next 5 columns where bold indicates which limb the participants agreed on most. (– indicates missing values). Numbers of evaluators per video are shown in the last column.
