# Supplementary material for: Rater agreement of visual lameness assessment in horses during lungeing
Source: Equine Vet J. 2015 Feb 2;48(1):78–82. doi: 10.1111/evj.12385 (PMC4964936; doi:10.1111/evj.12385)
Supplement: Supplementary file 3 — Supplementary Item 3: Number of videos (total 45) on horses with the different combinations of fore and hindlimb asymmetry categories during lungeing. [file EVJ-48-78-s003.pdf]

Equine Veterinary Journal

Supplementary Item 3: Number of videos (total 45) on horses with the different combinations of fore and hindlimb asymmetry categories during lungeing (2 videos excluded due to missing sensor data). No consideration has been taken to the direction of lungeing and possible circle-dependent asymmetries.

Hindlimb asymmetry	Forelimb asymmetry			
	**FL0** 0-6	**FL1** 6-12	**FL2** 12-18	**FL3** >18
**HL0** 0-3	3	1	1	6
**HL1** 3-6	6	0	0	5
**HL2** 6-9	3	0	1	3
**HL3** > 9	1	4	2	9
